# Modified unified critical state model for soils considering over-consolidation and cyclic loading behaviours

**DOI:** 10.1038/s41598-022-26624-x

**Published:** 2023-02-21

**Authors:** Xiaowen Wang, Ran Yuan, Kai Cui

**Affiliations:** 1 School of Civil Engineering, Southwest Jiaotong University, Chengdu, 610031 China; 2 Key Laboratory of Transportation Tunnel Engineering, Ministry of Education, Southwest Jiaotong University, Chengdu, 610031 China; 3 Key Laboratory of High-Speed Railway Engineering, Ministry of Education, Southwest Jiaotong University, Chengdu, 610031 China

**Keywords:** Civil engineering, Geology

## Abstract

This paper presents a modified unified critical state model to predict the mechanical responses of both clays and sands under over-consolidation and cyclic loading conditions on the basis of clay and sand model (CASM), which is named as CASM-kII. Through the application of subloading surface concept, CASM-kII is able to describe the plastic deformation inside the yield surface and the reverse plastic flow, and is thus expected to capture the over-consolidation and cyclic loading behaviours of soils. CASM-kII is numerical implemented by the using of the forward Euler scheme with automatic substepping and error control. Then, a sensitivity study is carried out to check the influences of the three new parameters of CASM-kII on the mechanical response of soils in over-consolidation and cyclic loading conditions. Through the comparisons of experimental data and simulated results, it is found that CASM-kII is able to satisfactorily describe the mechanical responses of both clays and sands in over-consolidation and cyclic loading conditions.

## Introduction

An accurate and concise description of the mechanical properties of soils is required for the design of civil engineering. As one of the most significant achievements in the field of geomechanics, Cam-clay plasticity^1^ has been well accepted for the constitutive modeling of soils^2–6^. In order to unify the constitutive modelling of both clays and sands, Yu^7^ developed a unified critical state model for clay and sand (i.e., CASM) on the basis of Cam-clay plasticity and state parameter concept. Owing to its concise mathematical expression and clear physical meaning of material constants, the CASM has been further developed to capture the more complex mechanical behaviours of soils^8–12^.

However, as stated by Hashiguchi^13–15^, classic elastoplastic constitutive models with a single yield surface enclosing the elastic domain possess many limitations in describing the mechanical response of soils. First, the original CASM predicts pure elastic behaviour inside the yield surface while the experimental data show that recoverable deformation only occurs within a very small range^16–19^. CASM describes discontinuous variation of the tangent stiffness modulus from elastic to plastic of over-consolidated soils and predicts an unsmooth stress–strain relation, and thus is unable to describe softening behaviour accurately^9,17,20^. Second, the ability to characterize the effect of stress history during cyclic loading process is significant to the constitutive modeling of soils^21–25^, since it has been discovered in cyclic loading tests that elastic and plastic deformations both develop during unloading before the stress path is completely reversed^26,27^. When the effect of reverse plastic flow is critical, the traditional isotropic hardening CASM model with a single yield surface is unable to provide appropriate solutions for boundary value problems because the reversed plastic flow fails to be considered just by expanding the single yield locus. Therefore, the above significant issues must be considered before further developing CASM to describe a variety of more complex properties of soils. Khong^9^ has attempted to introduce the concept of kinematic hardening into CASM within the context of bounding surface theory(i.e. CASM-k) but failed because of the difficulty of numerical implementation, thus Khong^9^ highly recommended that further development of CASM should be conducted in future research.

To extend the prediction ability of conventional elastoplastic constitutive model under complex loading conditions (such as over-consolidation, cyclic loading, anisotropic loading and non-proportional loading), Hashiguchi^13–15^ proposed the subloading surface concept with rigorous physical backgrounds. During last decades, subloading surface concept has been widely adopted in the constitutive modeling of soils. Hashiguchi et al.^17^ and Yao et al.^18^ adopted the subloading surface to describe the over-consolidated behaviour of soils. Asaoka et al.^28^ and Zhang et al.^29^ described the mechanical properties of structured soils through the combination of superloading and subloading surfaces. Since the concept of rotational hardening mechanism has been widely adopted in the constitutive modeling to capture the induced anisotropic properties of soils^30–32^, Hashiguchi and Chen^33^, Yamakawa et al.^34^ and Hashiguchi et al.^35^ combined the subloading surface concept with rotational hardening theory to characterize the cyclic mobility mechanism of anisotropic soils.

In this study, a new modified unified critical state model called CASM-kII is developed by introducing the subloading surface theory into the original CASM. This new model is established to extend the prediction ability of CASM for both clays and sands under over-consolidation and cyclic loading conditions. The plastic modulus of this new model is derived through the consistency condition of subloading function and varies smoothly from elastic to plastic response during loading procedure, which makes it possible for CASM-kII to characterize the plastic deformation inside the yield surface and the reverse plastic flow and thus to describe the over-consolidated behaviour and cyclic loading response of soils in a flexible fashion. Then, the forward Euler scheme with automatic substepping and error control is adopted in this study to numerical implement this newly proposed model. Compared with original CASM, CASM-kII adds three material parameters to control the evolution law of subloading surface, and the influences of these new parameters on the mechanical properties of soils under drained and undrained conditions is checked through a sensitivity study. At last, CASM-kII is validated through the comparisons of experimental data and simulation results in monotonic and cyclic loading tests under both drained and undrained conditions.

## Constitutive relation of CASM-kII

First, soil behaviour is assumed to be isotropic in this paper for the sake of simplicity. If the inherent and induced anisotropy behaviours of soils need to be considered, CASM-kII may be extended to account for anisotropy by incorporating anisotropic mechanism such as rotational hardening behaviour^30–35^ or soil fabric and its evolution rule^36–39^ in the model. The extension work for the issue of anisotropy can be referred to the work of Gao et al.^40^ Second, the bold-faced characters are used to represent vectors and tensors and the italics are used to represent scalars.

In this section, the subloading surface theory is introduced in CASM. The effective mean stress *p* and deviatoric stress *q* can be expressed as:

1 $$ p = \frac{1}{3} {\text{tr}} ({{\varvec{\upsigma}}}),\quad q = \sqrt {\frac{{3}}{{2}}} \left\| {{{\varvec{\upsigma}}} - p{{\varvec{\updelta}}}} \right\| $$

where ${{\varvec{\upsigma}}}$ is effective stress tensor; ${{\varvec{\updelta}}}$ is Kronecker delta.

**Figure 1:**
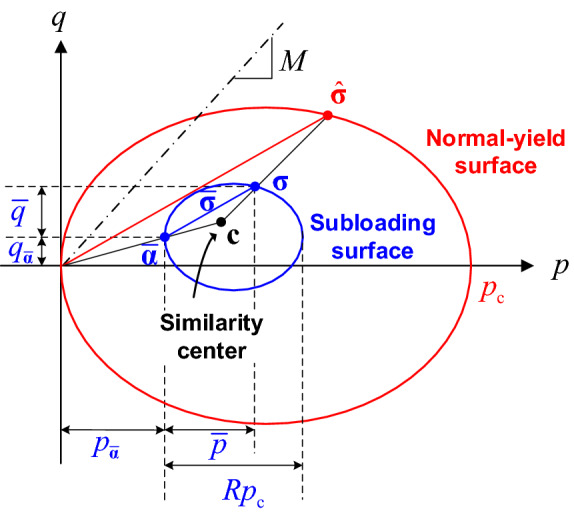
The relationship of subloading surface and normal-yield surface in *p* − *q* plane.

As illustrated in Fig. 1, it is assumed that a subloading surface exists inside the domain bounded by the typical yield surface (or called normal-yield surface), which indicates the loading history and represents the material's mechanical responses during the loading and unloading procedure^13–15^. The subloading surface passes always through the current stress state ${{\varvec{\upsigma}}}$, and approaches the normal-yield surface asymptotically in a plastic loading procedure. During the whole loading process, the shapes of subloading surface and normal-yield surface keep similar, and the normal-yield ratio is recorded as $R$. According to the geometric relationships shown in Fig. 1, the following relationships can be given as:

2 $$ \frac{{\left\| {{\overline{\mathbf{\sigma }}}} \right\|}}{{\left\| {{\hat{\mathbf{\sigma }}}} \right\|}} = \frac{{\overline{p}}}{{\hat{p}}} = \frac{{\overline{q}}}{{\hat{q}}} = \frac{{Rp_{{\text{c}}} }}{{p_{{\text{c}}} }} = R $$

3 $$ {\overline{\mathbf{\sigma }}} = {{\varvec{\upsigma}}} - {\overline{\mathbf{\alpha }}},\quad \overline{p} = p - p_{{{\overline{\mathbf{\alpha }}}}} ,\quad \overline{q} = q - q_{{{\overline{\mathbf{\alpha }}}}} $$

4 $$ {\overline{\mathbf{\alpha }}} = {\mathbf{c}} - R{\mathbf{c}} $$

5 $$ p_{{{\overline{\mathbf{\alpha }}}}} = \frac{{1}}{{3}}{\text{tr(}}{\overline{\mathbf{\alpha }}}{),}\quad q_{{{\overline{\mathbf{\alpha }}}}} = \sqrt {\frac{{3}}{{2}}} \left\| {{\overline{\mathbf{\alpha }}} - p_{{{\overline{\mathbf{\alpha }}}}} {{\varvec{\updelta}}}} \right\| $$

6 $$ \hat{p} = \frac{{1}}{{3}}{\text{tr(}}{\hat{\mathbf{\sigma }}}{),}\quad \hat{q} = \sqrt {\frac{{3}}{{2}}} \left\| {{\hat{\mathbf{\sigma }}} - \hat{p}{{\varvec{\updelta}}}} \right\| $$

where ${\overline{\mathbf{\alpha }}}$ denotes the position of subloading surface, $p_{{{\overline{\mathbf{\alpha }}}}}$ and $q_{{{\overline{\mathbf{\alpha }}}}}$ denote the projections of ${\overline{\mathbf{\alpha }}}$ on the *p* and *q* axes, respectively; ${\overline{\mathbf{\sigma }}}$ denotes the stress observed from subloading surface, $\overline{p}$ and $\overline{q}$ are the mean stress and deviatoric stress of ${\overline{\mathbf{\sigma }}}$, respectively; ${\hat{\mathbf{\sigma }}}$ denotes the conjugate stress of ${{\varvec{\upsigma}}}$ on the normal-yield surface, $\hat{p}$ and $\hat{q}$ are the mean stress and deviatoric stress of ${\hat{\mathbf{\sigma }}}$, respectively. For Cam-clay models, in particular, the size of normal-yield surface can be represented by $p_{{\text{c}}}$ (i.e., preconsolidation pressure), and the size of subloading surface can be represented by $Rp_{{\text{c}}}$.

### Functions of normal-yield surface and subloading surface

According to the work of Yu^7,20^, the stress-state relation for soils can be given as:

7 $$ \left( {\frac{\eta }{M}} \right)^{n} = {1} - \frac{\xi }{{\xi_{{\text{R}}} }} $$

8 $$ \xi = v + \lambda {\text{ln}}\,p - \Gamma $$

9 $$ \xi_{{\text{R}}} = {(}\lambda - \kappa {)ln}\,r $$

where $\eta = q{/}p$ is stress ratio, $\xi$ and $\xi_{{\text{R}}}$ are the state parameter and positive reference state parameter, respectively; $\lambda$, $\Gamma$ are the well-known Cam-clay parameters; $n$ and $r$ are the material parameters jointly controlling the shape of yield surface. $M$ denotes the slope of critical state line.

By substituting Eqs. (2)–(6) into Eq. (7), the normal-yield function $\hat{f}$ and subloading function $\overline{f}$ in general stress space can be defined as:

10 $$ \hat{f} = \left( {\frac{{\hat{q}}}{{M\hat{p}}}} \right)^{n} + \frac{{\ln \left( {\hat{p}{/}p_{{\text{c}}} } \right)}}{\ln \,r} = 0 $$

11 $$ \overline{f} = \left( {\frac{{q - q_{{{\overline{\mathbf{\alpha }}}}} }}{{M{(}p - p_{{{\overline{\mathbf{\alpha }}}}} )}}} \right)^{n} + \frac{{\ln \left[ {(p - p_{{{\overline{\mathbf{\alpha }}}}} )/Rp_{{\text{c}}} } \right]}}{\ln \,r} = 0 $$

It should be noted that subloading surface coincides with the normal-yield surface when *R* = 1. In this case, the subloading function becomes identical to the normal-yield function, and the subloading surface model exhibits a response similar to the conventional plasticity model.

### Elastic behaviour

In general stress space, the elastic stress–strain relation can be given as:

12 $$\Delta {{\varvec{\upsigma}}}{ = }{\mathbf{\rm E}}{:\Delta }{{\varvec{\upvarepsilon}}}^{{\text{e}}} { = }{\mathbf{\rm E}}{:(\Delta }{{\varvec{\upvarepsilon}}} -\Delta {{\varvec{\upvarepsilon}}}^{{\text{p}}} {)} $$

13 $$ {\mathbf{\rm E}}{ = }\left( {K - \frac{{2}}{{3}}G} \right){{\varvec{\updelta}}}{ \otimes }{{\varvec{\updelta}}} + 2G{\mathbf{\rm I}} $$

where $\Delta {{\varvec{\upsigma}}}$ is stress increment; $\Delta {{\varvec{\upvarepsilon}}}$, $\Delta {{\varvec{\upvarepsilon}}}^{{\text{e}}}$, $\Delta {{\varvec{\upvarepsilon}}}^{{\text{p}}}$ are the total, elastic and plastic strain increments, respectively; **E** denotes the elastic stiffness matrix; **I** is a fourth-order symmetric identity tensor; *K* and *G* denote the bulk and shear modulus, respectively. It should be noted that the elastic behaviour of granular materials is non-linear and depend on the stress level. There are two types of formulations are widely adopted to describe the non-linear elastic behaviour of granular materials, i.e., hypoelasticity^18,20,38,41,42^ and hyperelasticity^43–48^ formulations. Under the framework of hypoelasticity, *K* and *G* related to the mean stress *p* can be defined as:

14 $$ K = \frac{{v_{0} p}}{\kappa } $$

15 $$ G = \frac{3(1 - 2\mu )}{{2(1 + \mu )}}K $$

where $\kappa$ denotes the slope of swelling line in *v* *− *ln *p* space; $\mu$ denotes the Poisson’s ratio, $v_{0}$ denotes initial value of specific volume.

### Dilatancy rule

It is well known that the associated flow rule adopted by Cam-clay would lead to an over-prediction of shear strain for soils subjected to normal compression^49^. Therefore, the dilatancy rule of CASM-kII follows the relation suggested by Rowe^50^:

16 $$ D = \frac{{\Delta \varepsilon_{{\text{v}}}^{{\text{p}}} }}{{\Delta \varepsilon_{{\text{q}}}^{{\text{p}}} }} = \frac{{9(M - \overline{\eta })}}{{9 + 3M - 2M\overline{\eta }}} $$

where *D* is dilatancy rate; $\Delta \varepsilon_{{\text{v}}}^{{\text{p}}}$ and $\Delta \varepsilon_{{\text{q}}}^{{\text{p}}}$ are the volume component and shear component of plastic strain rate, respectively; $\overline{\eta } = \overline{p}/\overline{q}$ is the stress ratio on subloading surface.

### Plastic flow rule

The plastic potential function of subloading surface can be obtained by integrating the Rowe’s relationship:

17 $$ g = 3M\ln \frac{{\overline{p}}}{\beta } + (3 + 2M)\ln (2\overline{\eta } + 3) - (3 - M)\ln (3 - \overline{\eta }{)} = 0 $$

where $\beta$ is the size parameter.

Therefore, the plastic strain increment can be given as:

18 $$ \Delta {{\varvec{\upvarepsilon}}}^{{\text{p}}} = \Delta\Lambda {\overline{\mathbf{L}}} = \Delta\Lambda \frac{\partial g}{{\partial {\overline{\mathbf{\sigma }}}}} $$

where $\Delta\Lambda$ and ${\overline{\mathbf{L}}}$ denote the magnitude and direction of plastic strain increment, respectively. According to Eqs. (11) and (18), it should be noted that the yield properties and plastic flow of CASM-kII are applied to the subloading surface instead of the normal-yield surface, which is different from the traditional elastoplastic model.

### Hardening rule

**Figure 2:**
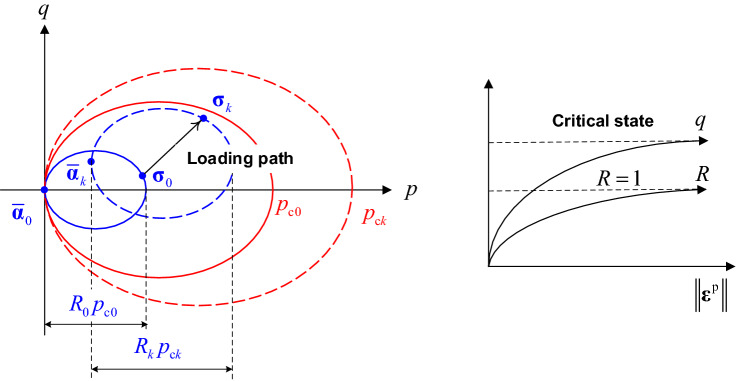
Schematic representation of the evolution law of subloading surface.

The hardening rule of the subloading surface are controlled by ${\overline{\mathbf{\alpha }}}$, *R* and $p_{{\text{c}}}$, and the isotropic hardening rule of the normal-yield surface is described by isotropic hardening parameter $p_{{\text{c}}}$, as illustrated in Fig. 2. The size of subloading surface is controlled by both $p_{{\text{c}}}$ and *R*, similar to the Cam-clay plasticity, the variable $p_{{\text{c}}}$ is controlled by the increment of plastic volumetric strain:

19 $$ \Delta p_{{\text{c}}} { = }\theta p_{{\text{c}}} \Delta \varepsilon_{{\text{v}}}^{{\text{p}}} ,\quad \theta = \frac{{v_{0} }}{{\lambda  - \kappa }} $$

20 $$ \Delta \varepsilon_{{\text{v}}}^{{\text{p}}} { = }\Delta {\Lambda} \hbox{tr} ({\overline{\mathbf{L}}}) $$

Integrating Eqs. (18) and (19) over a finite time increment yields the following alternative incremental hardening laws:

21 $$ p_{{{c}k + 1}} = p_{{{c}k}} \exp \left( {\theta \Delta \varepsilon_{{\text{v}}}^{{\text{p}}} } \right) $$

Considering that the stress approaches the normal-yield surface progressively, i.e., the subloading surface approaches the yield surface gradually during the plastic loading procedure, the rate of $R$ can be defined as:

22 $$ \Delta R = U\left\| {\Delta {{\varvec{\upvarepsilon}}}^{{\text{p}}} } \right\| = \Delta\Lambda U\left\| {{\overline{\mathbf{L}}}} \right\|\,,{\text{ when}}\,\Delta {{\varvec{\upvarepsilon}}}^{{\text{p}}} \ne {\mathbf{O}} $$

where ${\mathbf{O}}$ is a two-order zero tensor; *U* is a monotonically decreasing function of the normal-yield ratio *R*, and it must satisfy the following rules:

23 $$ \left\{ {\begin{array}{*{20}l} {U = + \infty } \hfill & {\text{for}} \hfill & {R = 0} \hfill \\ {U > 0} \hfill & {\text{for}} \hfill & {R \in (0,\,1)} \hfill \\ {U = 0} \hfill & {\text{for}} \hfill & {R = 1} \hfill \\ {U < 0} \hfill & {\text{for}} \hfill & {R > 1} \hfill \\ \end{array} } \right. $$

There are many mathematical expressions of *U* that can satisfy Eq. (23), and here we adopt a simple one:

24 $$ U = - h_{\text{m}} \ln (R) $$

where $h_{{\text{m}}}$ is a new material constant controlling the rate of subloading surface that approaches to the normal-yield surface.

It should be noted that during the elastic unloading process (the loading criterion will be given in the next section), *R* must be calculated according to the nonlinear Eq. (11) formed by current known values of ${{\varvec{\upsigma}}}$, ${\overline{\mathbf{\alpha }}}$ and $p_{{\text{c}}}$, because Eq. (22) only holds for the plastic loading process. However, the subloading equation is of high-order (*n*-order), which makes it impractical to obtain the analytical solution of *R*. Hence, the Newton–Raphson method might be appropriate for calculation of *R*. The complete calculation procedure of *R* during elastic unloading process is listed in Table 1.

**Table 1:** The numerical calculation procedure of *R* by the Newton–Raphson method.

Step	Brief description
1	Input the variables ${{\varvec{\upsigma}}}_{k}$, ${\mathbf{c}}_{k}$ and $p_{{{\text{c}}k}}$ at the end of step $k$
2	Set the initial value $R_{k}^{i} = R_{k}^{0} = R_{k + 1}$ and the tolerance error $TOL = 1{\text{e}} - 6$, where superscript $i$ indicates the number of iterations
3	Calculate the stress on the subloading surface and its components on the subloading surface ${\overline{\mathbf{\sigma }}}_{k} = {{\varvec{\upsigma}}}_{k} - (1 - R_{k}^{i} ){\mathbf{c}}_{k}$ $\overline{p}_{k} = \frac{1}{3}tr({\overline{\mathbf{\sigma }}}_{k} )$, $\overline{q}_{k} = \sqrt{\frac{3}{2}} \left\| {{\overline{\mathbf{\sigma }}}_{k} - \overline{p}_{k} {{\varvec{\updelta}}}} \right\|$
4	Compute the subloading surface function $\overline{f}_{k}^{i} = \left( {\frac{{\overline{q}_{k} }}{{M\overline{p}_{k} }}} \right)^{n} + \frac{{\ln (\overline{p}_{k} /R_{k}^{i} p_{{\text{c}k}} )}}{\ln r}$
5	Compute the derivative of subloading surface function with respect to $R_{k}^{i}$ $\frac{{\partial \overline{f}_{k}^{i} }}{{\partial R_{k}^{i} }} = n\frac{{\overline{q}_{k}^{n} }}{{M\overline{p}_{k}^{n + 1} }}(q_{{{\text{s}}k}} \overline{p}_{k} { - }p_{{{\text{s}}k}} \overline{q}_{k} ) + \frac{1}{\ln \,r}\left( {\frac{{p_{{{\text{s}}k}} }}{{\overline{p}_{k} }}{ - }\frac{1}{{R_{k}^{i} }}} \right)$ where $p_{{{\text{s}}k}} = \frac{1}{3}{\text{tr}}({\mathbf{c}}_{k} )$, $q_{{\text{s}k}} = \sqrt{\frac{3}{2}} \left\| {{\mathbf{c}}_{k} - p_{{\text{s}k}} {{\varvec{\updelta}}}} \right\|$
6	Update the normal-yield ratio by:$R_{k}^{i + 1} = R_{k}^{i} - \frac{{\overline{f}_{k}^{i} }}{{\partial \overline{f}_{k}^{i} /\partial R_{k}^{i} }}$
7	If $\left| {f_{k}^{i + 1} } \right| < TOL$, $R_{k} = R_{k}^{i + 1}$, and go to next step $k + 1$, else back to step 3

Besides, it should be noted that the similarity center should be located inside the normal-yield surface during the whole loading process. Following the concept of subloading surface, the similarity center surface can be defined as follows:

25 $$ f({\mathbf{c}}) = R_{{\text{cen}}} p_{{\text{c}}} $$

where the similarity center surface passes through the similarity center point and is similar to the normal-yield surface in terms of stress space origin. $R_{{{\text{cen}}}}$ is the ratio of the size of the similarity center surface to the normal-yield surface. To make certain that the similarity center lies inside the limit surface, i.e., $f({\mathbf{c}}) = r_{{\text{c}}} p_{{\text{c}}}$, the following inequality must hold:

26 $$ 0 \le f({\mathbf{c}}) \le r_{{\text{c}}} p_{{\text{c}}} ,\quad 0 \le R_{{{\text{cen}}}} \le r_{{\text{c}}} $$

where $r_{{\text{c}}}$ ($0 < r_{{\text{c}}} < 1$) is a new material constant designating the maximum value of $R_{{{\text{cen}}}}$. Besides, the similarity center surface is unable to be bigger than the normal-yield surface, by using the properties of homogeneous function, the closure condition of similarity center can be obtained:

27 $$ \frac{{\partial f({\mathbf{c}})}}{{\partial {\mathbf{c}}}}:\left[ {\Delta {\mathbf{c}}{ - }\frac{{\Delta p_{{\text{c}}} }}{{p_{{\text{c}}} }}{\mathbf{c}}} \right] \le 0,\,{\text{when}}\,0 \le R_{{\text{cen}}} \le r_{\text{c}} $$

Then, it can be assumed that the rate of similarity center can be written as:

28 $$ \Delta {\mathbf{c}} = \frac{{\Delta p_{{\text{c}}} }}{{p_{{\text{c}}} }}{\mathbf{c}} + h_{{\text{c}}} \left\| {\Delta {\varvec{\varepsilon}}^{{\text{p}}} } \right\|\left( {\frac{{{\overline{\mathbf{\sigma }}}}}{R} - \frac{{\mathbf{c}}}{{r_{\text{c}} }}} \right) $$

where $h_{{\text{c}}}$ is a new material constant controlling the evolution rate of similarity center.

By Substituting Eq. (28) into Eq. (4), the rate of ${\overline{\mathbf{\alpha }}}$ is given by:

29 $$ \Delta {\overline{\mathbf{\alpha }}} = (1 - R)\left\{ {h_{\text{c}} \left\| {\Delta {\varvec{\varepsilon}}^{{\text{p}}} } \right\|\left( {\frac{{{\overline{\mathbf{\sigma }}}}}{R} - \frac{{\mathbf{c}}}{{r_{{\text{c}}} }}} \right){ + }\frac{{\Delta p_{{\text{c}}} }}{{p_{{\text{c}}} }}{\mathbf{c}}} \right\} - {\mathbf{c}}\Delta R $$

### Consistency condition and plastic modulus

Different from the traditional elastoplastic model, the consistency condition of subloading surface model is applied to the subloading function instead of normal-yield function^13^. According to Eq. (11), the consistency condition of subloading function can be given as:

30 $$ {\overline{\mathbf{N}}}:\Delta {\overline{\mathbf{\sigma }}} + \overline{f}_{R} \Delta R + \overline{f}_{{p_{\text{c}} }} \Delta p_{\text{c}} = 0 $$

31 $$ {\overline{\mathbf{N}}} = \frac{{\partial \overline{f}}}{{\partial {\overline{\mathbf{\sigma }}}}},\quad \overline{f}_{R} = \frac{{\partial \overline{f}}}{\partial R},\quad \overline{f}_{{p_{\text{c}} }} = \frac{{\partial \overline{f}}}{{\partial p_{\text{c}} }} $$

Substituting Eqs. (12), (18), (19), (22) and (29) into Eq. (30), the plastic multiplier can be given as:

32 $$ \Delta \Lambda = \frac{{{\overline{\mathbf{N}}}:{\mathbf{E}}:\Delta {{\varvec{\upvarepsilon}}}}}{{K_{{\text{p}}} + {\overline{\mathbf{N}}}:{\mathbf{E}}:{\overline{\mathbf{L}}}}} $$

where $K_{{\text{p}}}$ is plastic modulus. According to the consistency condition of subloading function, the mathematical expression of $K_{{\text{p}}}$ is written as:

33 $$ K_{{\text{p}}} = {\overline{\mathbf{N}}}:(1 - R)\left\{ {\theta \text{tr}({\overline{\mathbf{L}}}){\mathbf{c}} + h_{{\text{c}}} \left\| {{\overline{\mathbf{L}}}} \right\|\left( {\frac{{{\overline{\mathbf{\sigma }}}}}{R} - \frac{{\mathbf{c}}}{{r_{\text{c}} }}} \right)} \right\} - {\overline{\mathbf{N}}}:{\mathbf{c}}U\left\| {{\overline{\mathbf{L}}}} \right\| + \frac{1}{\ln \,r}\left\{ {\frac{U}{R}\left\| {{\overline{\mathbf{L}}}} \right\| - \theta \,\text{tr}({\overline{\mathbf{L}}})} \right\} $$

By substituting Eq. (32) into Eq. (12), the elastoplastic stiffness matrix ${\mathbf{E}}^{{{\text{ep}}}}$ can be defined as:

34 $$ {\mathbf{E}}^{{{\text{ep}}}} = {\mathbf{E}} - \varsigma \frac{{{\mathbf{E}}:{\overline{\mathbf{L}}}{ \otimes }{\overline{\mathbf{N}}}:{\mathbf{E}}}}{{K_{{\text{p}}} + {\overline{\mathbf{N}}}:{\mathbf{E}}:{\overline{\mathbf{L}}}}} $$

where $\varsigma$ is the loading index, and the loading criterion^15^ is given as:

35 $$ \left\{ {\begin{array}{*{20}l} {{\overline{\mathbf{N}}}:{\mathbf{E}}:\Delta {{\varvec{\upvarepsilon}}} > 0,\, \varsigma = 1,} \hfill & {\text{Loading}} \hfill \\ {{\overline{\mathbf{N}}}:{\mathbf{E}}: \Delta {\varvec{\varepsilon}} \le 0, \, \varsigma = 0,} \hfill & {\text{Unloading}} \hfill \\ \end{array} } \right. $$

### Numerical implementation scheme

Existing numerical implementation schemes of the constitutive models are generally classified as backward Euler^51–54^ and forward Euler^55–57^ schemes. The backward Euler scheme with the return-mapping technique is accurate because the resulting stress automatically satisfy the yield function to a specified tolerance. However, this backward Euler method requires the second derivatives of the yield and the plastic potential functions, which makes it difficult to the implement for complex constitutive relations^58^. Compared with back Euler scheme, forward Euler scheme have the advantage of being more straightforward to implement. Therefore, the forward Euler scheme with automatic substepping and error control is adopted in this study because its simplicity and efficiency. The forward Euler scheme with automatic substepping and error control can divide the loading step into several substeps according to the local computational accuracy required. It is assumed that the strain increment input by the analysis system is $\Delta {{\varvec{\upvarepsilon}}}_{k + 1}$ at step $k$ to step $k + 1$, this original increment may be too large and leads to excessive error sometimes. To avoid the drift of yield surface and improve computational precision, it is necessary to divide this increment into a series of parts (substeps) to satisfy the local tolerance error. The complete load integration algorithm may be implemented as Table 2:

**Table 2:** Flowchart of the integration scheme.

Step	Brief description
1	Input initial variables ${{\varvec{\upsigma}}}_{k}$, ${{\varvec{\upvarepsilon}}}_{k}$, ${\mathbf{c}}_{k}$, $p_{{{\text{c}}k}}$, $R_{k}$ and the strain increment $\Delta {{\varvec{\upvarepsilon}}}_{k + 1}$
2	Set the pseudo time $T = 0$, $\Delta T = 1$ and the tolerance error $STOL = 1\text{e} - 6$
3	Calculate the first trial elasto-plastic stiffness matrix ${\mathbf{E}}_{1}^{{{\text{ep}}}} \left\{ {{{\varvec{\upsigma}}}_{k} , \, {\mathbf{c}}_{k} , \,p_{{{\text{c}}k}} , \, R_{k} } \right\}$ by substituting the initial variables into Eq. (34)
4	Compute the subincrement according to $\Delta {{\varvec{\upvarepsilon}}}_{k + 1}^{{\text{sub}}} = \Delta T \times \Delta {{\varvec{\upvarepsilon}}}_{k + 1}$
5	Calculate the first trial stress increment $\Delta {{\varvec{\upsigma}}}_{1} = {\mathbf{E}}_{1}^{{{\text{ep}}}} :\Delta {{\varvec{\upvarepsilon}}}_{k + 1}^{{{\text{sub}}}}$ and update the first trial state variables ${\mathbf{c}}_{1}$, $p_{{{\text{c1}}}}$ and $R_{1}$ related to plastic deformation
6	Compute the second trial elastoplastic stiffness matrix ${\mathbf{E}}_{2}^{{{\text{ep}}}} \left\{ {{{\varvec{\upsigma}}}_{1} ,\,{\mathbf{c}}_{1} ,\,p_{{\text{c}}}1 ,\, R_{1} } \right\}$ by substituting the first trial variables into Eq. (34)
7	Calculate the second trial stress increment $\Delta {{\varvec{\upsigma}}}_{2} = {\mathbf{E}}_{2}^{{{\text{ep}}}} :\Delta {{\varvec{\upvarepsilon}}}_{k + 1}^{{{\text{sub}}}}$
8	Calculate the average stress increment $\Delta {{\varvec{\upsigma}}}_{k + 1} = \frac{1}{2}(\Delta {{\varvec{\upsigma}}}_{1} + \Delta {{\varvec{\upsigma}}}_{2} )$
9	Compute the relative local error $LE = {{\left\| {\frac{1}{2}(\Delta {{\varvec{\upsigma}}}_{1} - \Delta {{\varvec{\upsigma}}}_{2} )} \right\|} \mathord{\left/ {\vphantom {{\left\| {\frac{1}{2}(\Delta {{\varvec{\upsigma}}}_{1} - \Delta {{\varvec{\upsigma}}}_{2} )} \right\|} {\left\| {{{\varvec{\upsigma}}}_{k} + \Delta {{\varvec{\upsigma}}}_{k + 1} } \right\|}}} \right. \kern-0pt} {\left\| {{{\varvec{\upsigma}}}_{k} + \Delta {{\varvec{\upsigma}}}_{k + 1} } \right\|}}$
10	If $LE > STOL$, the size of this substep is too large and a smaller pseudo time needs to be found: $\rho = \max \left\{ {0.9\sqrt {STOL/LE} ,\, 0.1} \right\}$, $\Delta T = \max \left\{ {\rho \Delta T, \, 0.001} \right\}$, then return to step 4 Else, go to next step
11	Compute the size of next substep $\rho = \min \left\{ {0.9\sqrt {STOL/LE} , \, 1.1} \right\}$ If the above equation is rejected, then $\rho = \min \left\{ {\rho ,\, 0.9} \right\}$ Update the pseudo time $T = T + \Delta T$, $\Delta T = \rho \Delta T$
12	Recalculate the pseudo time to ensure the size of next substep is bigger than that of minimum step and is not bigger than 1, the following conditions must be applied $\Delta T = \max \left\{ {\Delta T, \, 0.001} \right\}$, $\Delta T = \min \left\{ {\Delta T, \, 1 - T} \right\}$
13	While $T = 1$ end this iterative calculation and output the Jacobi stiffness matrix ${\mathbf{C}}_{k + 1}$ to the finite element routine for the global equilibrium iterations. The Jacobi stiffness matrix can be derived from the same procedure in solving the elastoplastic matrix^58^: ${\mathbf{C}}_{k + 1} = {\mathbf{E}}_{k + 1}^{{\text{ep}}} \left\{ {{{\varvec{\upsigma}}}_{k + 1} ,{\mathbf{c}}_{k + 1} ,p_{{\text{c}k + 1}} ,R_{k + 1} } \right\}$

## Model constants

The newly proposed model CASM-kII requires ten material parameters ($\Gamma$, $\lambda$, $\kappa$, $\mu$, $M$, $r$, $n$, $h_{{\text{m}}}$, $h_{{\text{c}}}$ and $r_{{\text{c}}}$) and initial values of the of the internal hardening variables ($p_{{\text{c}0}}$, $R_{0}$ and ${\overline{\mathbf{\alpha }}}_{0}$). The roles of each constant are listed in Table 3. $\Gamma$, $\lambda$, $\kappa$, $\mu$ and $M$ are the well-known constants in Cam-clay plasticity, which are called the “*basic constants*”. $r$ and $n$ are called yield constants because they control the shape of normal yield surface. The detailed calibration procedure of the basic and yield constants can be referred to the work of Yu^7,20^, Rios et al.^12^ and Navarro et al.^59^. $h_{{\text{m}}}$, $h_{{\text{c}}}$ and $r_{{\text{c}}}$ are the newly introduced material parameters used to control the evolution rule of subloading surface, which called “*Hardening constants*” in this study. The variable $p_{{{\text{c0}}}}$ can be determined by the preconsolidation pressure. As shown in Fig. 2, the initial value of the position tensor can be set as ${\overline{\mathbf{\alpha }}}_{0}$ = **O** for initial isotropy^15^. Once $p_{{{\text{c0}}}}$ and ${\overline{\mathbf{\alpha }}}_{0}$ are known, $R_{0}$ can be easily obtained from the subloading function of Eq. (11) as follows:

36 $$ R_{0} = \frac{{\overline{p}_{0} }}{{p_{{\text{c}0}} }}\exp \left[ {\left( {\frac{{\overline{q}_{0} }}{{M\overline{p}_{0} }}} \right)^{n} \ln r} \right] $$

where $\overline{p}_{0}$ and $\overline{q}_{0}$ are the initial mean stress and initial deviatoric stress on subloading surface, respectively.

**Table 3:** Model constants in CASM-kII.

Category	Symbol	Description
Basic constants	$\Gamma$	Intersection of critical state line with *p* = 1 kPa line in *e* – ln*p* space
	$\lambda$	Slope of compression line in *e* − ln*p* space
	$\kappa$	Slope of swelling line in *e* − ln*p* space
	$\mu$	The Poisson’s ratio
	$M$	Slope of critical state line in *p* − *q* space
Yield constants	$r$	Controls the shape of normal-yield surface
	$n$	Controls the shape of normal-yield surface
Hardening constants	$h_{{\text{m}}}$	Controls the evolution rate of the size of subloading surface
	$h_{{\text{c}}}$	Controls the evolution rate of the position of subloading surface
	$r_{{\text{c}}}$	Controls the maximum size of similarity center surface
Initial internal hardening variables	$p_{{{\text{c}}0}}$	Initial value of preconsolidation pressure
	$R_{0}$	Initial value of normal-yield ratio
	${\overline{\mathbf{\alpha }}}_{0}$	Initial position of subloading surface

As suggested by Hashiguchi^15^, $h_{{\text{m}}}$ can be determined from the stress–strain curve in the subyield state while $h_{{\text{c}}}$ and $r_{{\text{c}}}$ can be determined from the stress–strain curve in cyclic loading. Before using CASM-kII to predict the mechanical response of a practical engineering problem, it is instructive to investigate the influence of hardening constants on the predicted mechanical properties of soils under drained (or undrained) monotonic loading (or cyclic loading) conditions. In this study, a sensitivity study is carried out to check the influences of these three new parameters ($h_{{\text{m}}}$, $h_{{\text{c}}}$ and $r_{{\text{c}}}$) on the mechanical response of soils in over-consolidation and cyclic loading conditions. The basic and yield material constants are set as $\Gamma$ = 2.59, $\lambda$ = 0.15, $\kappa$ = 0.05, $\mu$ = 0.3, $M$ = 1, $r$ = 2.718, $n$ = 1.6 while the hardening parameters $h_{{\text{m}}}$, $h_{{\text{c}}}$ and $r_{{\text{c}}}$ are variables. It is assumed that the samples used in the test are isotropically consolidated with $p_{{{\text{c0}}}}$ = 100 kPa, $v_{0}$ = 2 and ${\overline{\mathbf{\alpha }}}_{0}$ = **O**. In the monotonic loading test under drained and undrained conditions, the OCRs of the samples are set to 4 and 8 ($R_{0}$ = 0.25 when OCR = 4, $R_{0}$ = 0.125 when OCR = 8). In the cyclic loading test under drained and undrained conditions, the samples are slightly over-consolidated (OCR = 1.5, $R_{0}$ = 0.667) and a two-way cyclic loading ($q$ =  ± 30 kPa) is applied.

**Figure 3:**
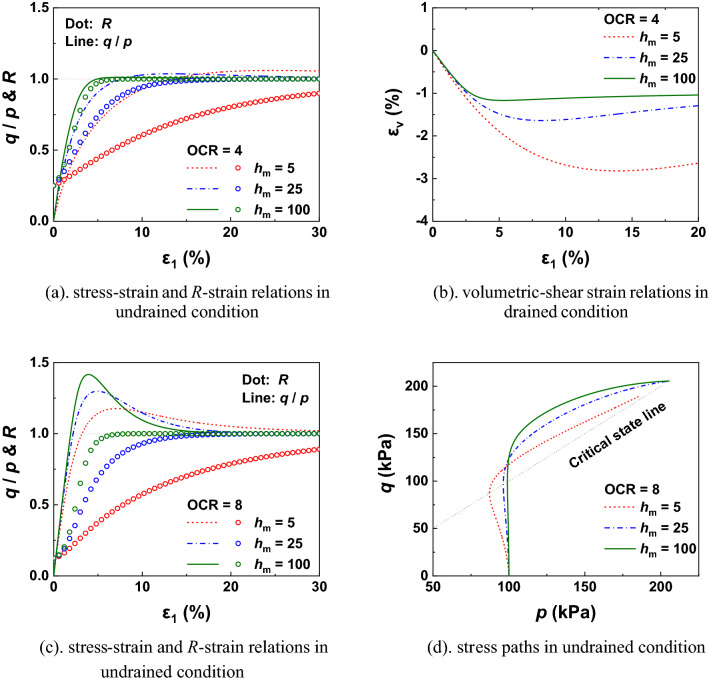
Drained and undrained simulations with different values of *h*_m_ in monotonic loading process.

As illustrated in Fig. 3, the value of $h_{{\text{m}}}$ significantly affects the mechanical properties (such as stress–strain relation, stress path, shear dilatancy and the normal-yield ratio evolution) of over-consolidated samples under monotonic loading. The sample reaches the critical state earlier (drained condition) and demonstrates a lager peak strength (undrained condition) with a bigger value of $h_{\text{m}}$ under plastic loading process.

**Figure 4:**
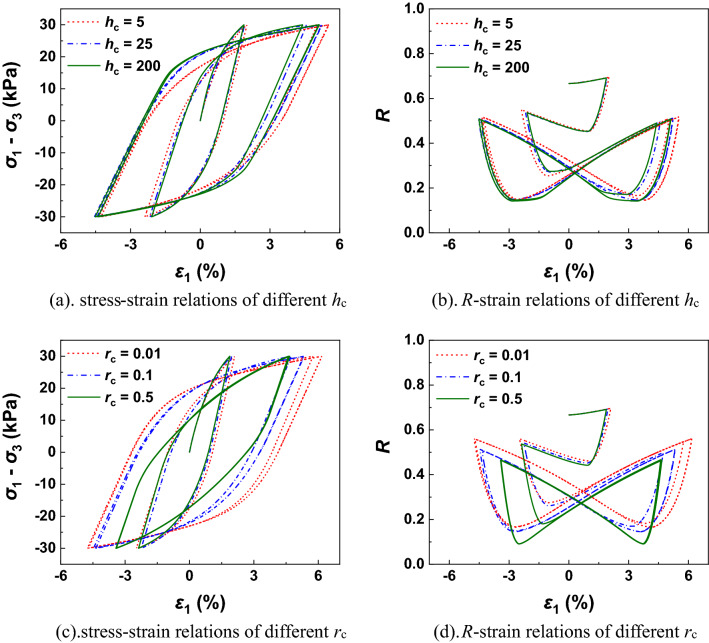
Undrained simulations with different values of *h*_c_ and *r*_c_ in cyclic loading process.

**Figure 5:**
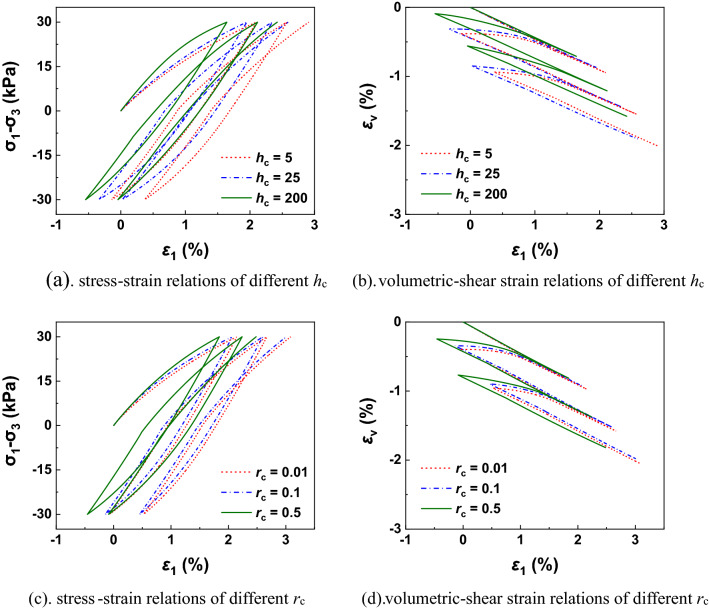
Drained simulations with different values of *h*_c_ and *r*_c_ in cyclic loading process.

Figure 4 shows the performances of different $h_{{\text{c}}}$ and $r_{{\text{c}}}$ under undrained cyclic loading tests. It can be found the hysteresis loop increases quicker (softening behaviour) with smaller values of $h_{{\text{c}}}$ and $r_{{\text{c}}}$. As illustrated in Fig. 4b,d, the normal-yield ratio *R* varies like a butterfly with the loading and unloading process. As shown in Fig. 5, the performances of different $h_{{\text{c}}}$ and $r_{{\text{c}}}$ under drained cyclic loading tests is checked. The accumulated plastic deformation and shear dilatancy under drained conditions demonstrate larger with smaller values of $h_{{\text{c}}}$ and $r_{{\text{c}}}$ because the rate of reverse plastic flow is slower at this time.

## Numerical simulations

In order to assess the performance of this newly proposed CASM-kII in monotonic and cyclic loading conditions, the experiment data of Jiangxi clay, Boston blue clay, Toyoura sand and Commercially available clay are used in this study. The values of the basic constants, yield constants and initial values of internal hardening variables used in the simulations are determined through the reported results and recommended values in previous study^11,21,60–62^, while the hardening parameters are determined through the methods in previous section. The material constants of these four samples are listed in Table 4.

**Table 4:** The material constants of different samples.

	Jiangxi clay	Boston blue clay	Toyoura sand	Commercially available clay
Basic constants	$\Gamma$ = 2.255	$\Gamma$ = 2.501	$\Gamma$ = 2.067	$\Gamma$ = 2.134
	$\lambda$ = 0.095	$\lambda$ = 0.184	$\lambda$ = 0.05	$\lambda$ = 0.173
	$\kappa$ = 0.018	$\kappa$ = 0.036	$\kappa$ = 0.0064	$\kappa$ = 0.05
	$\mu$ = 0.3	$\mu$ = 0.3	$\mu$ = 0.3	$\mu$ = 0.3
	$M$ = 1.36	$M$ = 1.35	$M$ = 1.3	$M$ = 0.772
Yield constants	$r$ = 2.2	$r$ = 2.718	$r$ = 12	$r$ = 2.718
	$n$ = 3.5	$n$ = 1.8	$n$ = 2	$n$ = 2
Hardening constants	$h_{{\text{m}}}$ = 95	$h_{{\text{m}}}$ = 40	$h_{{\text{m}}}$ = 80	$h_{{\text{m}}}$ = 750
	$h_{{\text{c}}}$ = 10	$h_{{\text{c}}}$ = 25	$h_{{\text{c}}}$ = 55	$h_{{\text{c}}}$ = 35
	$r_{{\text{c}}}$ = 0.95	$r_{{\text{c}}}$ = 0.9	$r_{{\text{c}}}$ = 0.7	$r_{{\text{c}}}$ = 0.95
Initial internal variables	$v_{0}$ = 1.88	$v_{0}$ = 2.01	$v_{0}$ = 1.71	$v_{0}$ = 2.15
	${\overline{\mathbf{\alpha }}}_{0} = {\mathbf{\rm O}}$	${\overline{\mathbf{\alpha }}}_{0} = {\mathbf{\rm O}}$	${\overline{\mathbf{\alpha }}}_{0} = {\mathbf{\rm O}}$	${\overline{\mathbf{\alpha }}}_{0} = {\mathbf{\rm O}}$
	OCR = 1:	OCR = 1:	$p$ = 1 MPa:	$p_{{\text{c}0}}$ = 450 kPa
	$p_{{\text{c}0}}$ = 98 kPa, $R_{0}$ = 1	$p_{{\text{c}0}}$ = 196 kPa, $R_{0}$ = 1	$p_{{\text{c}0}}$ = 8 MPa,	$R_{0}$ = 1
	OCR = 2:	OCR = 2:	$R_{0}$ = 0.125	
	$p_{{\text{c}0}}$ = 196 kPa, $R_{0}$ = 0.5	$p_{{\text{c}0}}$ = 392 kPa, $R_{0}$ = 0.5	$p$ = 2 MPa:	
	OCR = 8:	OCR = 8:	$p_{{\text{c}0}}$ = 10 MPa, $R_{0}$ = 0.2	
	$p_{{\text{c}0}}$ = 784 kPa, $R_{0}$ = 0.125	$p_{{\text{c}0}}$ = 1568 kPa, $R_{0}$ = 0.125	$p$ = 3 MPa: $p_{{\text{c}0}}$ = 10.5 MPa, $R_{0}$ = 0.286	

### Drained triaxial tests

**Figure 6:**
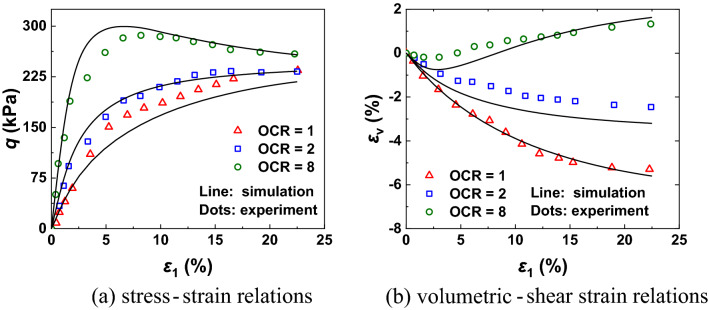
Model predictions and experiment results of Jiangxi clay under drained conditions.

The drained triaxial tests of Jiangxi clay was proposed by Hu et al.^62^ with these samples are applied consolidation confining pressure to 98 kPa, 196 kPa and 784 kPa in triaxial apparatus. These samples had a height of 80 mm and a diameter of 39.1 mm. In order to prepare samples with over consolidation ratios of 1, 2 and 8, the confining pressure was unloaded to 98kpa step by step. As illustrated in Fig. 6, CASM-kII can accurately simulate the stress–strain and deformation characteristics in normally and over-consolidation conditions. In particular, the newly proposed model is found to be relatively capable of capturing the dilatancy behaviour of the overconsolidated clay observed in the laboratory tests.

### Undrained triaxial tests

**Figure 7:**
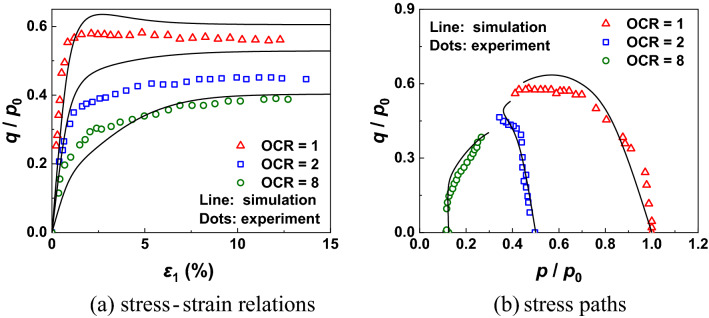
Model predictions and experiment results of Boston blue clay under undrained conditions.

The measured data of Boston blue clay of Pestana et al.^60^ is used here to validate the prediction ability of CASM-kII for the mechanical properties of overconsolidated clays under undrained loading process, with the pre-consolidation pressure set to 196 kPa and the over-consolidation ratios set to OCR = 1, 2, 8. Figure 7 shows that the agreement between measured data and simulation data of both stress–strain relation and stress paths is satisfactory. Besides, CASM-kII is able to predict the smooth stress–strain curves in over-consolidation conditions, whilst the original CASM fails to predict^9^.

**Figure 8:**
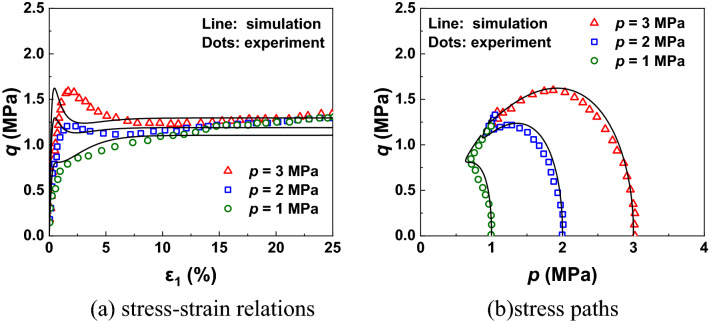
Model predictions and experiment results of Toyoura sand under undrained conditions.

Then, the experimental data of Toyoura sand observed by Verdugo and Ishihara^63^ is used here to evaluate the prediction ability of CASM-kII to capture the behaviour of sands. The undrained tests of Toyoura sand were conducted under very high confining pressures (*p* = 1, 2, 3 MPa) with same void ratio *e*_0_ = 0.71. The initial values of internal variables can be referred to the work of Zhang et al.^61^. As shown in Fig. 8, the results simulated by CASM-kII coincide well with the experimental results quantitatively and qualitatively. Under the same void ratio, the sands behave the properties of loose sands when the confining stress is large, while the sands behave the properties of dense sands when the confining stress is small. Such a phenomenon is called as “*confining-stress dependency of sand*”^64^.

### Cyclic loading tests

**Figure 9:**
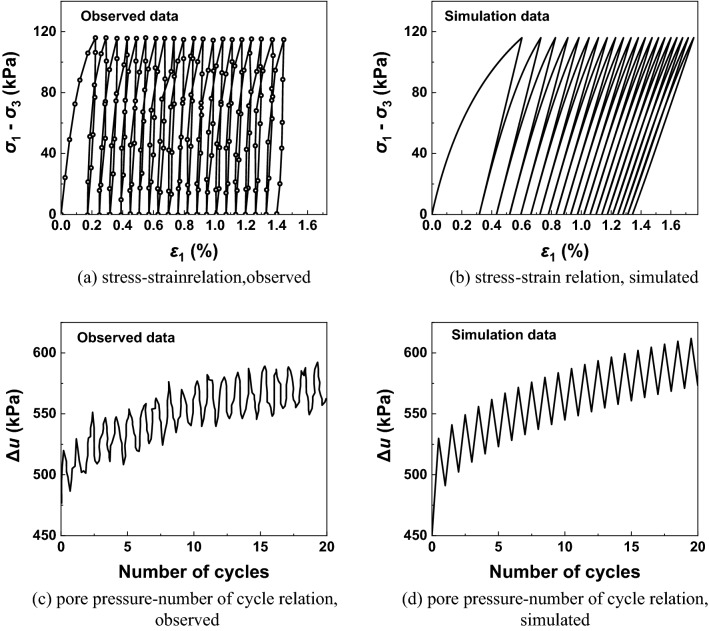
Model predictions and experiment results of Commercially available clay under undrained conditions.

To verify the applicability of the new model under cyclic loading conditions, the experiment data observed by Li and Meissner^21^ is used here. The material constants and initial conditions of these samples can be found in Table 4. These cyclic tests are stress-controlled with the half amplitude of the deviatoric stress *q* = 116 kPa. Figure 9 shows the observed data and the simulation data, the deviatoric stress is plotted against the axial strain while the excess pore water pressure ($\Delta {\varvec{u}}$) is plotted against the number of cycles, where CASM-kII can describe the behaviour of undrained commercially available clay under cyclic loading conditions.

## Conclusions

In this paper, a modified unified critical state model (called CASM-kII) to predict the mechanical properties of both clays and soils under over-consolidation and cyclic loading conditions is developed on the basis of original CASM model and subloading surface concept. Through the introduction of subloading surface, this newly proposed model is able to describe the plastic deformation inside the yield surface and the reverse plastic flow, and is thus expected to accurately capture the over-consolidated and cyclic behaviours. CASM-kII is numerical implemented by the using of the forward Euler scheme with automatic substepping and error control. Then, a sensitivity study is carried out to check the influences of these three new parameters ($h_{\text{m}}$, $h_{\text{c}}$ and $r_{\text{c}}$) on the mechanical response of soils in over-consolidation and cyclic loading conditions. Through the comparisons of experimental data and simulated results, it is found that CASM-kII performs well in the simulations of both clays and sands in over-consolidation and cyclic loading conditions.

## Data Availability

Some or all data, models, or code that support the findings of this study are available from the corresponding author upon reasonable request.
